# Clinical, pathological characteristics, and therapeutic outcomes of primary ovarian carcinoid tumors: a case series of 15 cases

**DOI:** 10.1186/s12957-025-03731-3

**Published:** 2025-03-11

**Authors:** Xinyue Dai, Suidan Chen, Simeng Yang

**Affiliations:** 1 Department of Gynecology, The First Affiliated Hospital of Wenzhou Medical University, No.2, Fuxue Lane, Lucheng District, Wenzhou, 325000 Zhejiang China; 2 Department of Pathology, The First Affiliated Hospital of Wenzhou Medical University, Wenzhou, 325000 Zhejiang China

**Keywords:** Ovarian neoplasms, Carcinoid tumor, Immunohistochemistry, Prognosis, Case series

## Abstract

**Background:**

The exact characteristics of primary ovarian carcinoid tumors remain largely unknown because of the rarity of the cases. This study aimed to investigate the clinical features, pathological characteristics, and therapeutic outcomes of patients with primary ovarian carcinoid tumors.

**Methods:**

This retrospective case series included patients with primary ovarian carcinoid tumors diagnosed between January 2009 and December 2023 at the First Affiliated Hospital of Wenzhou Medical University.

**Results:**

Fifteen patients were included. They were 45.8 ± 2.7 years at diagnosis. Eight tumors were located in the left ovary, while seven were in the right. All patients were stage I. Microscopically, nine tumors were classified as strumal carcinoid, two as insular carcinoid, three as trabecular carcinoid, and one as mixed. Synaptophysin (Syn) was positive in 14 cases, chromogranin A (CgA) in 10, CD56 in eight, thyroid transcription factor 1 (TTF-1) in five, and thyroglobulin (TG) in six. Twelve patients had a Ki67 index ≤ 7%. All 15 patients underwent surgery, with eight retaining fertility. Among them, one patient underwent comprehensive staging surgery, four underwent lateral adnexectomy, and three underwent cyst stripping. Seven patients underwent total hysterectomy and bilateral adnexectomy, including two patients undergoing comprehensive staging surgery. Three patients received intravenous chemotherapy. One patient was lost to follow-up. The remaining patients were followed up for 48–148 months; they were without recurrences and alive at the last follow-up.

**Conclusions:**

Primary ovarian carcinoid tumors present with atypical symptoms and signs. Surgical intervention may be an optimal choice for treatment, leading to favorable prognostic outcomes.

## Background

Most carcinoid tumors are typically found in the bronchopulmonary and gastrointestinal tracts [1]. Primary ovarian carcinoid tumors constitute an exceptionally rare subset of ovarian neoplasms, accounting for fewer than 1% of ovarian malignancies and only 0.5-1.7% of all carcinoid tumors [2, 3]. According to an early study, primary ovarian carcinoid tumors occur mainly in perimenopausal or menopausal women but can also occur in premenopausal women; among the 50 patients, only one died from the disease [4].

As delineated in the 5th edition of the World Health Organization (WHO) Classification of Tumors of the Female Reproductive System, primary ovarian carcinoid tumors can be categorized based on histopathological features into four distinct types: insular, trabecular, strumal, and mucinous [5]. The strumal variant is characterized by a variable admixture of carcinoid elements and thyroidal tissue, with either an intermixed or distinctly delineated arrangement. In such neoplasms, the thyroidal component exhibits immunoreactivity for thyroid transcription factor-1 (TTF-1) and thyroglobulin (TG), whereas these markers are not typically expressed in the carcinoid component [5, 6].

The exact characteristics of such tumors remain largely unknown because of the rarity of the cases [7]. Indeed, most available literature is restricted to one or two cases [8–11]. The largest series, comprising 50 cases, was published in 1980 [4]. Therefore, this study aimed to investigate the clinical features, pathological characteristics, and therapeutic outcomes of patients diagnosed with primary ovarian carcinoid tumors.

## Methods

### Study design and patients

This retrospective case series included all consecutive patients diagnosed with primary ovarian carcinoid tumors at the First Affiliated Hospital of Wenzhou Medical University between January 2009 and December 2023, all confirmed by pathological examination. Initially, 18 cases were identified, but three patients were excluded due to incomplete clinicopathologic records. During the study period, primary ovarian carcinoid tumors accounted for approximately 0.8% of all ovarian malignancies diagnosed at our institution. The inclusion criterion was a diagnosis of a primary ovarian carcinoid tumor established at the study hospital according to the 5th edition of the WHO Classification of Tumors of the Female Reproductive System [5]. Patients with missing comprehensive clinicopathologic records, except for postoperative immunohistochemistry (IHC), were excluded. This study was approved by the Ethics Committee of Clinical Research of the First Affiliated Hospital of Wenzhou Medical University (KY2023-R234). The requirement for informed consent was waived by the committee.

### Data collection

The demographic and clinical information (including age, reproductive history, history of prior gynecologic conditions, clinical manifestations, and findings), imaging test results (including gynecological ultrasonography and comprehensive abdominal computed tomography (CT)), and laboratory investigations (including tumor markers like CA125, CA19-9, carcinoembryonic antigen, and α-fetoprotein), treatment approaches, surgical pathology, chemotherapy, and postoperative follow-up information were extract from the patient records and hospital systems.

The surgical strategies included fertility-sparing procedures (i.e., unilateral oophorectomy, salpingo-oophorectomy, and comprehensive staging operations involving removal of the affected adnexa) and procedures not preserving fertility (i.e., total hysterectomy with bilateral salpingo-oophorectomy, and complete staging for ovarian malignancies).

The pathological examination included intraoperative frozen sections, postoperative pathology, and IHC examinations using the EnVision two-step technique, with a focus on key markers such as synaptophysin (Syn), chromogranin A (CgA), cluster of differentiation 56 (CD56), Ki67, TG, and TTF-1. Due to the study’s retrospective nature, these markers were not necessarily assessed in all patients. The study pathologist reviewed the slides according to the 2020 WHO Classification Criteria for Tumors of the Female Reproductive System [5].

Follow-up data were collected from the patient charts and ended on December 31, 2023. These assessments focused on the patient’s overall health status and any signs of recurrence or metastasis. If a patient had no clinical event, follow-up was censored on the date of the last patient contact. If the patient had a clinical event (recurrence or death), follow-up was terminated when the patient showed a predetermined endpoint.

### Statistical analysis

Data analysis was performed using SPSS 25.0 (IBM Corp., Armonk, NY, USA). Only descriptive statistics were used. The categorical variables were presented as n (%). The continuous variables were presented as mean ± standard deviation.

## Results

### Characteristics of the patients

The study included 15 patients. The mean age at presentation was 45.8 ± 2.7 years (range: 26 to 66 years). The average reproductive history included 0–5 pregnancies and 0–3 deliveries. Fourteen (93.3%) patients presented with pelvic masses at physical examination, with 13 exhibiting no obvious clinical symptoms and one reporting lower abdominal pain. One (6.7%) patient had increased menstrual flow due to multiple leiomyomas. Elevated levels of CA125 were observed in two (13.3%) cases, two (13.3%) patients had elevated CA199, and individual cases exhibited elevated AFP, CEA, and CA724 levels. The tumors were located in the left ovary in eight (53.3%) patients and the right ovary in seven (46.7%) patients. Tumor size varied from 1.5 to 15 cm (maximum diameters). The patients were stage Ia (*n* = 13) or Ic (*n* = 2) (Table 1).

**Table 1:** Clinicopathologic characteristics and therapeutic outcomes

Case	Age (years)	Side	Primary clinical manifestations	Overhaul	Tumor size (cm)	teratoma	Accompanying tumors	Tumor stage	Surgical procedures	Complementary therapy	Follow-up (months)
1	57	Right	PE revealed pelvic masses	Cystic	3 × 3; 2 × 2	No	No	Ia	Total hysterectomy and double adnexa	Radiotherapy	148
2	33	Right	PE revealed a pelvic mass	Cystic-solid	12 × 10 × 8	Yes	No	Ic	Right adnexectomy + greater omentectomy + pelvic lymph node dissection + pelvic adhesion release	Radiotherapy	146
3	49	Left	PE revealed pelvic masses	Cystic	12 × 8 × 13	Yes	Mucinous cystadenoma	Ia	Total hysterectomy and double adnexa + greater omentum + appendix + radical pelvic lymph node dissection	Radiotherapy	130
4	46	Right	PE revealed pelvic masses	Cystic	5 × 4 × 4; 1.5 × 1 × 1	Yes	No	Ia	Total hysterectomy and double adnexa	No	128
5	62	Left	PE revealed a pelvic mass	Cystic-solid	Diameter 8	Yes	No	Ia	Total hysterectomy and double adnexa	No	123
6	48	Right	PE revealed a pelvic mass	Cystic	3 × 3 × 2	No	No	Ia	Right Tubo-Ovariectomy	No	an unauthorized visit
7	34	Left	PE revealed a pelvic mass	Cystic	Diameter 7	Yes	No	Ia	Left ovarian cyst removed	No	102
8	41	Left	PE revealed a pelvic mass	Cystic-solid	12 × 10	No	No	Ic	Left salpingo-oophorectomy	No	94
9	48	Left	Increased menstrual flow for 2 years	Solid	4 × 3	No	No	Ia	Total hysterectomy and double adnexa	No	91
10	46	Right	PE revealed a pelvic mass	Cystic	1.5 × 1.0 × 1.0	Yes	No	Ia	Total hysterectomy and double adnexa + radical pelvic lymph node dissection + partial omentectomy + peritoneal biopsy	No	88
11	48	Left	PE revealed a pelvic mass	Cystic	5 × 4 × 4	No	No	Ia	Left ovarian cyst removed	No	84
12	42	Right	More than 3 years of lower abdominal pain lasting for 12 h and PE revealed a pelvic mass.	Cystic	15 × 10 × 10	No	No	Ia	Right tubo-ovariectomy	No	66
13	41	Left	PE revealed a pelvic mass	Solid	1.5 × 1	No	No	Ia	Left salpingo-oophorectomy	No	56
14	26	Left	PE revealed a pelvic mass	Cystic	8 × 7	No	No	Ia	Left ovarian cyst excision	No	51
15	66	Right	PE revealed a pelvic mass	Cystic-solid	5 × 4	No	No	Ia	Total hysterectomy and bilateral salpingo-oophorectomy	No	48

PE, physical examination

### Intraoperative and postoperative pathological examination

Intraoperative frozen section analysis was performed in 13 cases: five (33.3%) were diagnosed as carcinoid, three (20.0%) as gonadal-stromal, two (13.3%) as mature teratoma, one (6.7%) as struma ovarii, one (6.7%) as a teratoma with carcinomatous transformation (evidenced by a papillary thyroid carcinoma component within the cystic teratoma), and one (6.7%) as a mucinous cystadenoma with areas of adenocarcinoma.

According to the macroscopic examination, the tumors displayed diverse morphologies: nine (60.0%) were purely cystic, filled with yellow, greasy fluid and occasionally hair, four (26.7%) were cystic-solid, predominantly cystic with fluid content and occasionally exhibiting grease and hair on the cyst wall, and two (13.3%) were solid with greyish-white section surfaces.

The tumors were classified as strumal carcinoid (*n* = 9), insular carcinoid (*n* = 2), trabecular (*n* = 3), and mixed (*n* = 1); none were mucinous carcinoid tumors. Concomitant ovarian tumor components were observed in eight (53.3%) cases, with one case presenting both an ovarian teratoma and a mucinous cystadenoma and seven cases featuring an ovarian teratoma alone. The concordance rate between the frozen section and final paraffin-embedded pathology was five out of 13 cases.

**Fig. 1:**
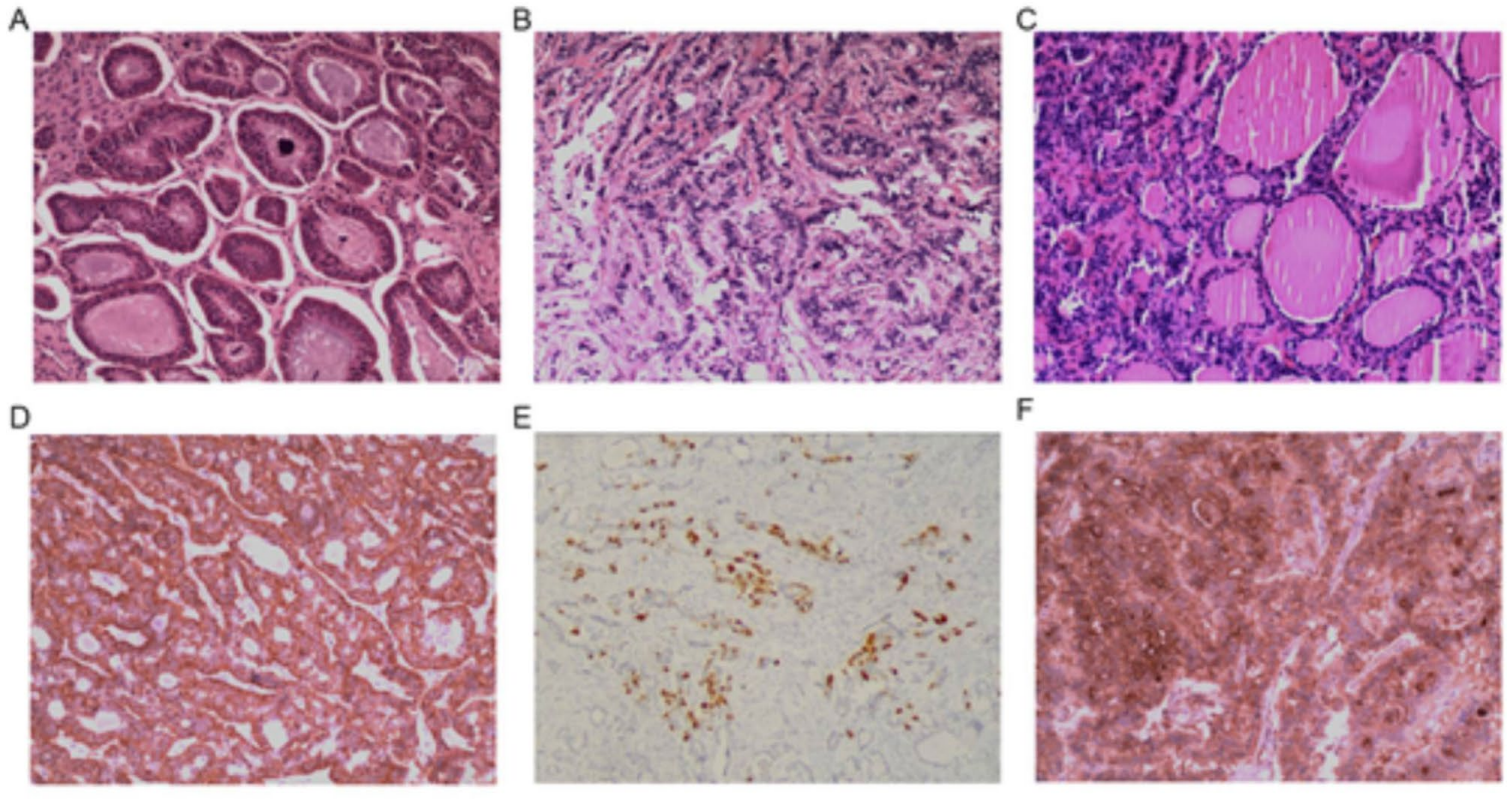
Postoperative pathological and immunohistochemical results. (**A**) Case #1, insular carcinoid tumor (hematoxylin & eosin staining, 100×). (**B**) Case #4, trabecular carcinoid tumor (hematoxylin & eosin staining, 100×). (**C**) Case #11, strumal carcinoid tumor (hematoxylin & eosin staining, 100×). (**D**) Case #7, positive synaptophysin staining in carcinoid tumors (hematoxylin & eosin staining, 100×). (**E**) Case #12, chromogranin A positivity in carcinoid tumors (hematoxylin & eosin staining, 100×). (**F**) Case #7, strumal carcinoid tumor (hematoxylin & eosin staining, 100×)

Histologically, the carcinoid component cells were small and uniform, with some arranged in acinar or trabecular patterns and abundant cytoplasm. Nuclei were round or oval with homogeneous staining, and nuclear pleomorphism was infrequent. Insular carcinoid tumors consisted of cellular nests and follicles, with small vesicles or round/oval islands of solid cell clusters composed of round or polygonal cells featuring oval or round nuclei (Fig. 1A). The cells of trabecular carcinoid tumors were predominantly arranged in parallel, beam-like structures or cords and embedded within a dense fibrous stromal matrix. The elongated, columnar tumor cells were oriented perpendicularly to the trabeculae, with uniformly sized, oval, or round nuclei (Fig. 1B). Strumal carcinoids exhibited a combination of insular and/or trabecular patterns with an ovarian struma component, with the two elements being intermixed or intermingled (Fig. 1C).

IHC analysis was conducted on the specimens from 14 patients (Table 2). Syn was universally positive across all 14 cases. CgA was positive in 10 (66.7%) cases and negative in four (26.7%). The Ki67 index varied: 1% in five (33.3%) cases, < 1% in five (33.3%), 7% in one (6.7%), and < 5% in one (6.7%) (two cases were not evaluated). CD56 was positive in eight (53.3%) cases, negative in one (6.7%), and equivocal in one (6.7%). Thyroid tissue within strumal carcinoids was positive for both thyroid TTF-1 and TG, whereas the carcinoid component did not express these markers. Of the nine cases of strumal carcinoid tumors, four were positive for both TTF-1 and TG, two were solely positive for TG, and one for TTF-1 only (the remaining two cases were not tested). The immunophenotypic profile was positive for Syn, CgA, and TG (Fig. 1D-F).

**Table 2:** Pathologic subtypes and IHC analysis

		Immunohistochemical indicators and intensity					
Case	Pathological subtypes	Syn	CgA	CD56	Ki67	TG	TTF-1
1	Insular carcinoid	+	+	–	1%+	Unchecked	Unchecked
2	Mixed carcinoid	+	Isolate **+**	Unchecked	Unchecked	Unchecked	Unchecked
3	Trabecular carcinoid	+	–	Unchecked	1%+	Unchecked	Unchecked
4	Trabecular carcinoid	No IHC	No IHC	No IHC	No IHC	No IHC	No IHC
5	Strumal carcinoid	+	Portion +	+	< 1%+	Unchecked	Unchecked
6	Strumal carcinoid	+	Sparse +	+	Unchecked	–	Thyroid tissue +
7	Strumal carcinoid	+++	–	+	< 1%+	Thyroid tissue +	Unchecked
8	Trabecular carcinoid	++++	++++	Unchecked	< 5%+	Unchecked	Unchecked
9	Insular carcinoid	+	–	+/-	7%+	Unchecked	Unchecked
10	Strumal carcinoid	+	+	+	< 1%+	Thyroid tissue +	–
11	Strumal carcinoid	+	–	+	About 1%+	Thyroid tissue +	Thyroid tissue +
12	Strumal carcinoid	+	Handful +	Portion +	1%+	Thyroid tissue +	Thyroid tissue +
13	Strumal carcinoid	+	Isolate **+**	Unchecked	1%+	Thyroid tissue +	Thyroid tissue +
14	Strumal carcinoid	+	+	+	< 1%+	Unchecked	Unchecked
15	Strumal carcinoid	+	+	+	< 1%+	Thyroid tissue +	Thyroid tissue +

Syn: synaptophysin; CgA: chromogranin A; CD56: cluster of differentiation 56; TG: thyroglobulin; TTF-1: thyroid transforming factor 1; IHC: immunohistochemistry

### Treatment and follow-up

All 15 patients underwent surgical intervention, with eight (53.3%) patients undergoing fertility-sparing procedures: one underwent comprehensive staging surgery with removal of the affected adnexa, four had the affected adnexa resected, and three had only the affected cysts excised. Among patients older than 45 years, seven underwent total hysterectomy with bilateral adnexectomy, two of which were part of comprehensive staging procedures.

Adjuvant chemotherapy was administered to three patients. One patient received three cycles of the BEP regimen (bleomycin, cisplatin, and etoposide) but did not complete treatment due to chemotherapy-induced myelosuppression. One patient was treated with one cycle of BVP (bleomycin, vincristine, and cisplatin) followed by three cycles of BEP. One patient received two cycles of TP (paclitaxel and carboplatin) but discontinued treatment due to myelosuppression. Hence, 12 patients underwent surgery alone.

The median follow-up was 92.5 months (range: 48 to 148 months) (Table 1). One patient was lost to follow-up due to incorrect contact information. All patients were alive and free from recurrence at the last follow-up. One patient successfully conceived after cancer treatments and delivered successfully.

## Discussion

This retrospective study investigated the clinical features, pathological characteristics, and therapeutic outcomes of patients diagnosed with primary ovarian carcinoid tumors. The results suggested that primary ovarian carcinoid lacks typical clinical symptoms and signs. IHC can help with the histopathological diagnosis, particularly Syn positivity. Treatment primarily involved surgery, and the patients had a favorable prognosis. Hence, considering that most ovarian tumors are aggressive and require extensive interventions, successfully identifying primary ovarian carcinoid tumors would be important to avoid overtreatment.

Primary ovarian carcinoid tumors are uncommon, comprising less than 0.1% of all ovarian malignancies [3], which represent 1.6% of all cancers [12], primarily affecting perimenopausal women [4]. An analysis of international cancer databases by Nasioudis et al. [13] revealed a mean age at diagnosis of 51.5 years, with most cases being stage I. In the present study, all patients were stage I (two with stage Ic disease and 13 with stage Ia), with a mean age of 45.8 ± 2.7 years, approximately 5 years younger than previously reported, possibly influenced by sample size and ethnicity. This study contributes to the limited body of literature on primary ovarian carcinoid tumors by providing a detailed analysis of clinical, pathological, and therapeutic outcomes in a relatively large case series. While our findings align with previous reports, the rarity of these tumors necessitates larger, multicenter international studies to further elucidate their characteristics and optimize treatment strategies.

The patients in this study were mostly asymptomatic, with pelvic masses discovered incidentally during physical examinations. The definitive diagnosis of primary ovarian carcinoid tumors relied on postoperative pathological evaluation. Among the 15 patients, 87% reported no significant clinical symptoms, while one presented with abdominal pain and another with menorrhagia due to multiple uterine leiomyomas. Carcinoid syndrome, estimated to occur in roughly 14% of patients with primary ovarian carcinoid tumors [14], is associated with symptoms such as facial flushing, diarrhea, bronchospasm, and carcinoid heart disease. Robboy & Scully [4] reported that 34% of their patients were asymptomatic, while 54% reported symptoms related to the tumor size, including abdominal swelling, pain, and constipation.

The histopathological classification of primary ovarian carcinoid tumors comprises various subtypes, including insular carcinoid tumors, trabecular carcinoid tumors, mixed insular and trabecular carcinoid tumors, strumal carcinoid tumors, and the exceedingly rare mucinous carcinoid tumors [15, 16]. Within this spectrum, strumal (goiter) carcinoid tumors emerge as the predominant subtype. These neoplasms rarely occur in isolation and are frequently associated with other ovarian pathologies, such as teratomas and mucinous cystadenomas. The literature supports that over half of primary ovarian carcinoid cases feature a teratomatous component [14]. Consistent with this, eight of the 15 cases reported here had concurrent ovarian tumor components. One case exhibited both ovarian teratoma and mucinous cystadenoma, while seven harbored only ovarian teratoma.

Given that the diagnostic focus for ovarian teratoma typically revolves around the identification of immature neural elements or germ cell tumor components, concomitant carcinoid tumors can be inadvertently overlooked. This oversight is especially pronounced during intraoperative frozen section examinations, as evidenced by the low concordance rate (38%) with the final pathological examination observed in the present study. Meticulous examination is warranted for all teratoma specimens to mitigate this diagnostic gap. Sending sections from irregular capsule walls or papillary regions for frozen analysis may be prudent to exclude the presence of carcinoid tumors and prevent missed diagnoses.

IHC is a crucial diagnostic tool for primary ovarian carcinoid tumors, with tumor cells typically demonstrating argentaffin characteristics. Neuroendocrine markers such as Syn, CgA, and neuron-specific enolase (NSE) have proven valuable in diagnostic practices [17]. In the present study, Syn was expressed in all tested cases. CgA was positive in most cases, albeit with lower intensity and distribution compared to Syn. These findings align with the literature [6, 18]. Syn and CgA are classical neuroendocrine markers in the diagnosis of primary ovarian carcinoid tumors. CD56 is also documented as a marker for these tumors [19], but it lacks specificity. Nonetheless, CD56 was detected in many patients in the present study (80%, 8/10). Furthermore, thyroid tissue within strumal carcinoid tumors expresses TTF-1 and TG, whereas carcinoid tissue lacks these markers [20]. Consistently, among nine cases of strumal carcinoid tumors reported here, four were positive for TTF-1 and TG, two for TG alone, and one for TTF-1 alone (two cases were not tested). Ki67 is an index of cellular activity and proliferation and has been reported to be low in primary ovarian carcinoid tumors, with values not exceeding 5% [9, 21]. In the present series, all 11 cases evaluated for Ki67 exhibited indices below 5%, except for one instance that reached 7%, underscoring the low malignant potential of this tumor type. In addition, the low levels of Ki-67 are indicative of a favorable prognosis [9, 22]. While TTF1 and caudal type homeobox 2 (CDX2) can assist in distinguishing between primary and metastatic carcinoid tumors, the distinction is not always marked [23].

The primary treatment for ovarian carcinoid tumors is surgery [24]. When the intraoperative frozen pathological examination of a unilateral ovarian tumor indicates a carcinoid tumor, exploration of the contralateral ovary and small intestine is imperative to ascertain the site of the primary tumor. The presence of a mass in the small intestine and mesentery, coupled with bilateral ovarian involvement, suggests a diagnosis of metastatic ovarian carcinoid tumor.

Patients with early-stage primary ovarian carcinoid tumors exhibit excellent survival outcomes. Notably, individuals with smaller tumors (diameter < 4 cm) have a survival rate exceeding 95%, and the overall survival rate for stage I patients appears unaffected by hysterectomy [13]. Total abdominal hysterectomy with bilateral adnexectomy is acknowledged as an acceptable treatment for these tumors [6], with age being a key determinant in the decision for hysterectomy. All 15 patients survived until the last follow-up visit. However, due to the small sample size, it was not possible to determine the impact of tumor size and surgical approach on prognosis, but the available literature suggests that hysterectomy does not impact long-term prognosis in stage I patients [13]. The role of appendectomy in the management of primary ovarian carcinoid tumors remains controversial, although it is commonly performed in cases of mucinous ovarian carcinoid tumors [14]. The literature suggests better overall survival in stage I patients than in stage II-IV ones [13].

A case report described treatment with paclitaxel and gemcitabine in a patient with recurrent primary ovarian mucinous carcinoid tumor after surgery, exhibiting disease stability during treatment but progression after chemotherapy discontinuation, highlighting the chemoresistance of ovarian carcinoid tumors [25]. In the present study, three patients with early-stage disease received adjuvant chemotherapy; the regimens varied, with one receiving platinum and paclitaxel and the others undergoing germ cell tumor-targeted BVP and BEP chemotherapy. Due to the limited number of cases and follow-up duration, whether early-stage patients may benefit from adjuvant chemotherapy remains unknown, and the question warrants further investigation [26].

The present study highlights the importance of carefully evaluating ovarian tumors. Primary ovarian carcinoid tumors have a favorable prognosis compared with typical ovarian cancers [27], an accurate identification is necessary to avoid overtreatment. Nevertheless, despite their good prognosis, early-stage ovarian carcinoid tumors can present complications such as carcinoid syndrome and carcinoid disease, warranting thorough clinical evaluation and sustained follow-up [28].

The study had several limitations. First, the small sample size of 15 cases from a single center limits the generalizability of the findings. Second, there is a risk that cases misdiagnosed as other types of ovarian tumors were not included, as they could not be identified from the databases. Third, the retrospective nature of the study restricted the data to what was available in the medical charts, potentially leading to incomplete or inaccurate information. Future multicenter studies with larger sample sizes are needed to further elucidate the clinical and pathological characteristics of primary ovarian carcinoid tumors and to validate the findings presented here.

## Conclusions

In conclusion, this retrospective study of 15 cases provides insights into the clinical features, pathological characteristics, and therapeutic outcomes of primary ovarian carcinoid tumors. Despite their rarity and atypical presentation, a careful pathological examination, including IHC, is crucial for accurate diagnosis. Surgical intervention remains the cornerstone of treatment, and in this series, patients generally experienced favorable prognoses. However, due to the limited sample size, these findings should be interpreted with caution, and larger, multicenter studies are needed to further validate these results.

## Data Availability

All data generated or analyzed during this study are included in this article.

## Abbreviations

CgA: Chromogranin A
TTF-1: Thyroid transcription factor 1
TG: Thyroglobulin
WHO: World Health Organization
IHC: Immunohistochemical
CT: Computed tomography
Syn: Synaptophysin
CD56: Cluster of differentiation 56
NSE: Neuron-specific enolase
CDX2: Caudal type homeobox 2
